# Predicting potential transmission risk of Everglades virus in Florida using mosquito blood meal identifications

**DOI:** 10.3389/fepid.2022.1046679

**Published:** 2022-12-02

**Authors:** Kristin E. Sloyer, Narayani Barve, Dongmin Kim, Tanise Stenn, Lindsay P. Campbell, Nathan D. Burkett-Cadena

**Affiliations:** ^1^ Department of Entomology & Nematology, Florida Medical Entomology Laboratory, Institute of Food and Agricultural Sciences, University of Florida, Vero Beach, FL, United States; ^2^ Department of Ecology & Evolutionary Biology, University of Tennessee, Knoxville, TN, United States

**Keywords:** Everglades virus, *Culex cedecei*, *Sigmodon hispidus*, ecological niche models, bloodmeal analysis

## Abstract

The overlap between arbovirus host, arthropod vectors, and pathogen distributions in environmentally suitable habitats represents a nidus where risk for pathogen transmission may occur. Everglades virus (EVEV), subtype II Venezuelan equine encephalitis virus (VEEV), is endemic to southern Florida where it is transmitted by the endemic vector *Culex cedecei* between muroid rodent hosts. We developed an ecological niche model (ENM) to predict areas in Florida suitable for EVEV transmission based upon georeferenced vector-host interactions from PCR-based blood meal analysis from blood-engorged female *Cx. cedecei* females. Thirteen environmental variables were used for model calibration, including bioclimatic variables derived from Daymet 1 km daily temperature and precipitation values, and land use and land cover data representing percent land cover derived within a 2.5 km buffer from 2019 National Land Cover Database (NLCD) program. Maximum temperature of the warmest month, minimum temperature of the coldest month, and precipitation of the driest month contributed 31.6%, 28.5% and 19.9% to ENM performance. The land cover types contributing the greatest to the model performance were percent landcover of emergent herbaceous and woody wetlands which contributed 5.2% and 4.3% to model performance, respectively. Results of the model output showed high suitability for *Cx. cedecei* feeding on rodents throughout the southwestern portion of the state and pockets of high suitability along the northern east coast of Florida, while areas with low suitability included the Miami-Dade metropolitan area and most of northern Florida and the Panhandle. Comparing predicted distributions of *Cx. cedecei* feeding upon rodent hosts in the present study to historical human cases of EVEV disease, as well as antibodies in wildlife show substantial overlap with areas predicted moderate to highly suitable for these vector/host associations. As such, the findings of this study likely predict the most accurate distribution of the nidus of EVEV to date, indicating that this method allows for better inference of potential transmission areas than models which only consider the vector or vertebrate host species individually. A similar approach using host blood meals of other arboviruses can be used to predict potential areas of virus transmission for other vector-borne diseases.

## Introduction

The overlap between arbovirus host and vector distributions in environmentally suitable habitats can provide useful information toward understanding where risk for pathogen transmission to humans or domestic animals may occur. Landscape epidemiology is a sub-discipline of landscape ecology which studies dynamic interactions between pathogens, hosts, and vectors across heterogeneous environments at multiple spatiotemporal scales (1, 2). The formal concept outlining associations between vector-borne disease and specific landscape features or habitat types was initially proposed by Pavlovsky (3) who suggested that vector-borne pathogen transmission could only occur in environments which support the overlap of three critical biological elements, including: competent vectors, vertebrate hosts, and the pathogen. Together, these components are often referred to as a nidus, or an area where pathogen transmission takes place (2, 3).

Ecological niche modeling (ENM) is used to predict potential geographic distributions of species by correlating environmental values with georeferenced occurrence points for model calibration (4). In the study of vector-borne diseases, ENMs have been used to predict distributions of vectors (5, 6), vertebrate hosts (7) and pathogens (8, 9), based upon georeferenced detections of each independent aspect of the nidus. Ecological niche models predicting the potential distribution of vectors, hosts, or pathogens are typically generated separately. While important, these single-species ENMs provide potential distributions for just one component of the nidus of pathogen transmission, a practice that is likely to overestimate the areas where pathogen transmission occurs. Although methods are available that can test whether environmental similarities exist in predicted distributions across two species (10–12), overlap is not evidence of interactions (typically blood-feeding) between vectors and hosts. Furthermore, separate models may not use the same covariates in model calibration, introducing an added challenge to making predictions across broader geographic areas. Similarly, several factors could affect the interactions between vector, host, and pathogen, such as the presence of dilution hosts which may draw vector species away from potential reservoir hosts (13), which are not accounted for when modeling disease system components individually. Therefore, a niche model using presence data available for known interactions (actual contact through blood-feeding) of two or more components of the nidus overlap should constitute a more accurate approach to predicting areas of potential pathogen transmission than modeling just vector, host, or pathogen distributions alone.

Everglades virus (EVEV) is an enzootic subtype of Venezuelan equine encephalitis virus (VEEV) and is maintained between the enzootic vector mosquito and rodent hosts in southern Florida (14). *Culex cedecei* is the only confirmed natural vector of EVEV in Florida, and feeds to a large extent on rodents, especially the hispid cotton rat (*Sigmodon hispidus* Say and Ord) (15–18). For this reason, interactions between *Cx. cedecei* and the hispid cotton rat are generally considered to be the primary drivers of EVEV transmission (16, 19). However, several other muroid rodent species are the natural hosts of EVEV and other enzootic subtypes of VEEV distributed throughout the Americas (14, 20). This is supported by the detection of substantial virus titers for VEEV subtypes from *Rattus* spp. in Colombia (21), along with high seroprevalence for EVEV in cotton mice (*Peromyscus gossypinus* Le Conte) and hispid cotton rats in Florida (22–25). *Culex cedecei* has also been found to feed heavily on black rats (*Rattus rattus* Linnaeus), cotton mice, and hispid cotton rats in areas where EVEV has been detected from mosquitoes in nature (17), providing evidence that all three rodent species are capable of developing viremias high enough for the transmission of EVEV to *Cx. cedecei* in Florida. Everglades virus can therefore be considered a single vector system, with few host species, wherein only rodent hosts and the mosquito vector are necessary to maintain pathogen transmission (12).

In the case of EVEV, modeling only the hosts would not provide a distribution representative of the risk of EVEV, but could greatly overestimate EVEV risk due to the ability of the three primary rodent hosts to utilize a range of habitat types (26, 27) and their wide geographical distributions. Likewise, although the distribution of the vector species *Cx. cedecei* may be more limited in its utilization of habitats and known distribution, modeling only the distribution of this species may also result in over-estimation of the potential distribution of EVEV foci if *Cx. cedecei* does not feed on EVEV hosts throughout its range, or across all types of climate and land cover habitats. It is also not straightforward to model the distribution of incidence of disease in humans, as these infections may not accurately represent the locations where they contracted the pathogen. Sloyer et al. (2022) has previously modeled the potential distributions of *Cx. cedecei* in Florida using environmental variables which include temperature, precipitation, and enhanced vegetation index (EVI). Results showed that the potential distribution of *Cx. cedecei* ranges from low to high suitability throughout much of the southern half of the Florida Peninsula. This model however did not take into account landscape composition and configuration and also cannot inform on whether or not *Cx. cedecei* feeds upon rodent hosts of EVEV in all areas. Aside from direct observations, and baited traps, the most common method of determining mosquito-host interactions is by performing polymerase chain reaction (PCR)-based blood meal analysis from blood-engorged mosquitoes to determine the host species origin of the blood meal (28, 29).

The objective of this study was to use geographically referenced blood meals of *Cx. cedecei* to generate an ENM using climate and landscape variables to predict suitable environments for interactions between *Cx. cedecei* and rodent hosts of EVEV in Florida. Resulting outputs generated by the ENM predict areas suitable for *Cx. cedecei* to feed on rodent hosts. We then overlay EVEV infections from previous studies in humans (30–33), wildlife (24, 25, 34, 35), and dogs (36) to illustrate how our model corresponds to natural infections in vertebrate animals. This method allows for the generation of a model representing the interaction between two components of the nidus of pathogen transmission rather than a single component, making it potentially more informative for inferring areas of potential transmission of EVEV.

## Materials and methods

### Data collection and preparation

**Figure 1:**
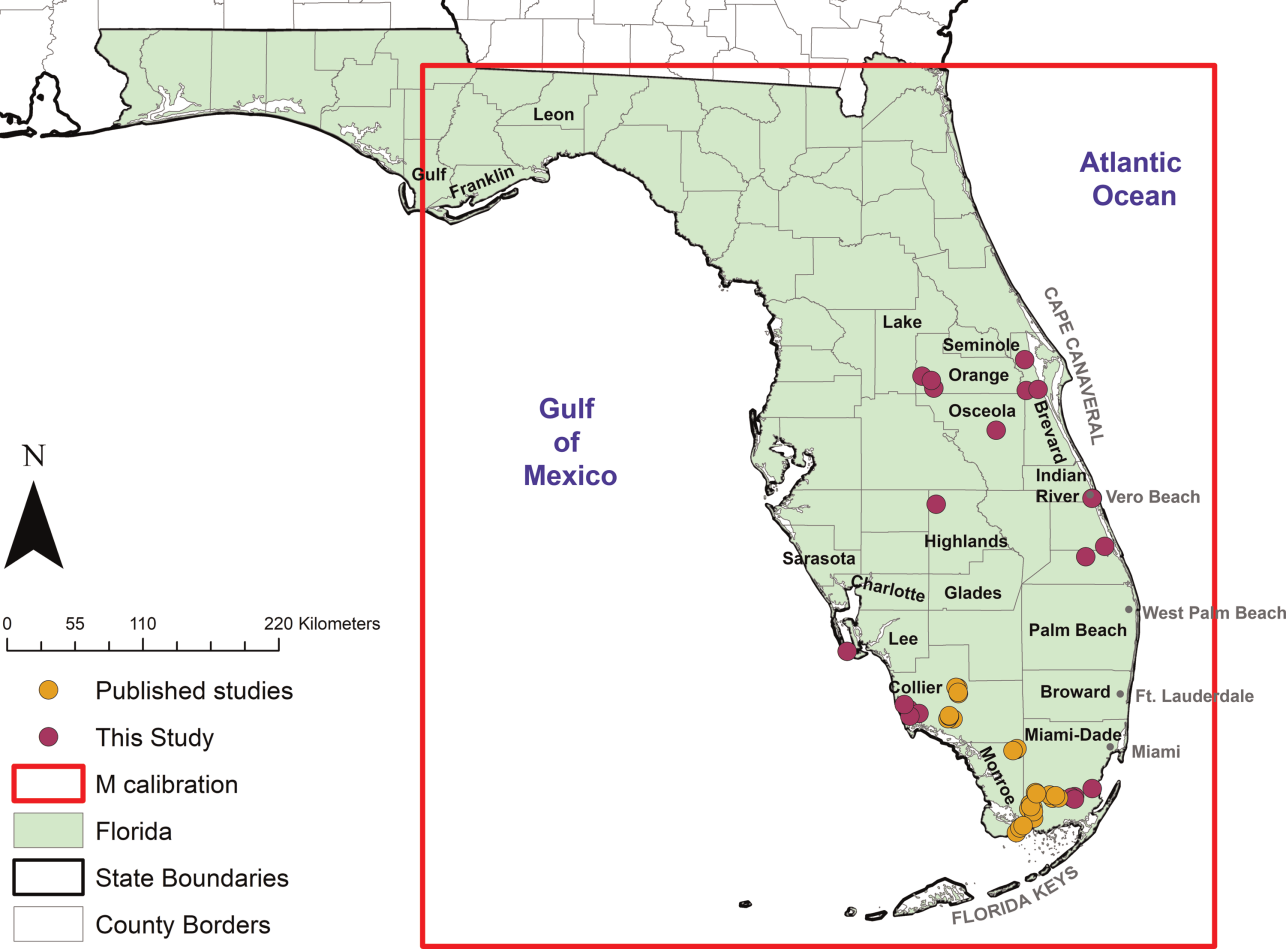
Georeferenced occurrence points (orange circles) used in the model representing *Cx. cedecei* rodent blood meals from this study and previous publications (16–18).

Georeferenced *Cx. cedecei* and rodent host interactions derived from blood meals were compiled from a combination of targeted field collections and previously published data (16–18). In both targeted field collections and published data, blood-engorged *Cx. cedecei* were collected using resting shelters, as previously described (37). Targeted data collection for this study occurred from the central counties of Orange and Brevard to Miami-Dade and Collier counties in the south (Figure 1). Previously published data of blood-engorged *Cx. cedecei* were processed and collected in a similar manner to those collected for the present study and described in greater detail therein (Supplementary Table S1) (16–18).

Vertebrate host identifications were determined using PCR-based blood meal analysis of blood-engorged *Cx. cedecei*. For samples collected for this study, blood-engorged mosquitoes were stored on dry ice or in a −80 freezer until samples were processed. To process blood meals, engorged abdomens of *Cx. cedecei* were smeared onto Whatman Flinders Technology Associates (FTA) cards to preserve host DNA (38). For this, small sections of the blood samples of FTA cards were cut out using box-cutter razor blades, disposing of contaminated blades between each sample, and placed into 1.5 ml micro centrifuge tubes with five to ten glass beads for DNA extraction. DNA from blood-engorged *Cx. cedecei* was extracted using InstaGene™ Matrix (Catalog #: 732-6030; Bio-Rad Laboratories, Inc., Hercules, CA, USA) using a previously described protocol (39, 40). Host DNA was amplified from the extracted product using primers which target the 16s rRNA and cytochrome *b* genes as described in previous publications of mosquito host-use (16, 18, 41). Samples were screened using two 16s rRNA targeting primers including 16L1/H3056 for reptiles and amphibians, and L2513/H2714 for mammals (42, 43). The primer pair L0/H1, which targets the same region of the cytochrome *b* gene was used for species-level identification of birds (44). Polymerase chain reaction cycling conditions were identical to those previously published (40, 41). Successful amplicon products were sent to Eurofins Genomics for Sanger sequencing and resulting sequences were identified to vertebrate host species using the National Center for Biotechnology Information (NCBI) GenBank Basic Local Alignment Search Tool (BLAST) (39).

Georeferenced occurrence points for vector-rodent interactions were defined as one of three rodent species blood meals from the primary enzootic vector *Cx. cedecei* from field sites in Florida, USA. The three rodent species chosen to build the model included hispid cotton rat, cotton mouse, and black rat based on potential to develop infectious titers of VEEV subtypes as reported in host competence studies and viral isolations (19, 21, 25, 45, 46). Sequences for *Rattus* spp. (*R. rattus* and *Rattus norvegica* Berkenhout) were highly similar (99% similarity). For this analysis, we consider *Rattus* spp. blood meals to originate from *R. rattus* (black rat) due to a small but consistently higher percent match in NCBI BLAST. After combining a total of 49 positive georeferenced rodent blood meals from this study and previous studies, the data were thinned spatially within a 0.001 degree distance (∼1 km), which removed 1 record, with 48 occurrence points remaining for model calibration (Figure 1).

### Model calibration area

We defined our model calibration area using the *M*-calibration region described in (47, 48). We limited the calibration area based on the known and modeled distributions of *Cx. cedecei* in Florida, including all of Peninsular Florida, the Florida Keys, and part of the Florida Panhandle, as previously described by Sloyer et al. (6). The calibration region for this model extends further north and west than the current known distributions of *Cx. cedecei* in Florida.

### Environmental data

Bioclimatic variables derived from Daymet 1 km daily temperature and precipitation values from 2010 to 2020 (49) using the “bioclim” function in the “dismo” package in R served as climate variables in the model (50). National Land Cover Database (NLCD) from 2019 served as the land use and land cover data in the model (51). Percent land cover representing landscape composition, and edge density representing landscape configuration were calculated within a 2.5 km distance and aligned to the bioclimatic variables using custom R code, allowing us to combine these data in the same model and predict across new locations.

The bioclimatic and landscape variables were checked for multicollinearity to identify variable candidate sets, as correlation between layers can contribute to difficulty in model interpretation (52). To check for multicollinearity, bioclimatic and landscape variable raster data were first masked to the M-calibration region and the variance inflation factor (VIF) values were calculated using the “vif” function in the “usdm” package in R (53). Four bioclimatic variables and percent land cover and edge density for ten NLCD habitat classes were checked for multicollinearity with a threshold value >5.

Two candidate sets of climatic and landscape variables were compiled based on their putative suitability to predict suitable habitat for *Cx. cedecei* and rodent species which include the hispid cotton rat, cotton mouse, and black rat, based on published habitat observations or recorded host-utilization by *Cx. cedecei* (6, 17, 26, 27, 54).

### Model calibration and evaluation

Models were run in the ENMeval v.2.0.0 package in R using the “glmnet” algorithm (55, 56). Georeferenced occurrence data was partitioned into training and testing data internally using the “ENMevaluate” function with a spatial block method to help reduce potential bias from spatial autocorrelation. Candidate models were generated separately for the two candidate sets including either edge density or percent land cover values using all combinations of the following feature classes: “linear”, “product”, “quadratic”, “linear + product”, “quadratic + product”, “linear + quadratic”, and “linear + product + quadratic”. Regularization multiplier values ranged sequentially between 1 and 4. Models were evaluated using an information criterion approach, ranking the models from lowest to highest Akaike's information criterion score (AIC) values and observing differences between the AUC train and test values (57). The best performing model was then run in Maxent (58) using the same parameter settings, but running with 50 bootstrapped replicates, and no extrapolation or clamping to produce variable response curves. Models were then projected to a broader area across Florida using the default “cloglog” output.

## Results

Results of the blood meal analyses on mosquitoes captured with resting shelters were categorized as vector-host interactions and georeferenced to provide occurrence records for ecological niche models. These models were used to predict where suitable habitat for *Cx. cedecei* and EVEV rodent hosts (cotton rats, cotton mice, and black rats), may be located, in order to predict areas where higher potential EVEV transmission may occur in Florida. A total of 175 blood engorged *Cx. cedecei* were collected and processed as a part of the present study. Of these, 150 blood meals (85.7%) were successfully identified to vertebrate host species. There were an additional five host species identifications which were identified from multiple host species blood meals, accounting for a total of 155 vertebrate host identifications. Vertebrate hosts included 13 species of mammal comprising 95.5% of identifications, four species of birds (3.9% of all host identifications), and one reptile host (Table 1). From the 155 host identifications, 68 (43.9%) were found to be derived from one of the three rodent hosts, with 22 of the 68 rodent host identifications from unique locations and used in the model (Table 1). Another 27 georeferenced rodent blood meals were compiled from previously published studies (16–18).

**Table 1:** Number and percent of total of host blood meals by host-class use and species use for *Cx. cedecei* in Florida.

Host class	Common name	Latin name	Blood-meals (*n*)	Percent of total (%)
Mammal			**148**	**95** **.** **5**
	Nine-banded armadillo	*Dasypus novemcinctus*	6	3.9
	Black rat	*Rattus rattus*	9	5.8
	Dog	*Canis lupus familiaris*	1	0.6
	Rabbit	*Sylvilagus* spp.	34	21.9
	Eastern woodrat	*Neotoma floridana*	10	6.5
	Hispid cotton rat	*Sigmodon hispidus*	49	31.6
	Human	*Homo sapiens*	1	0.6
	Cotton mouse	*Peromyscus gossypinus*	10	6.5
	Raccoon	*Procyon lotor*	7	4.5
	River otter	*Lontra canadensis*	1	0.6
	Virginia opossum	*Didelphis virginiana*	15	9.7
	White-tailed deer	*Odocoileus virginianus*	4	2.6
	Wild boar	*Sus scrofa*	1	0.6
Bird		* *	**6**	**3** **.** **9**
	Chicken	*Gallus gallus*	3	1.9
	Common yellowthroat	*Geothlypis trichas*	1	0.6
	Green heron	*Butorides virescens*	1	0.6
	Loggerhead shrike	*Lanius ludovicianus*	1	0.6
Reptile		* *	**1**	**0** **.** **6**
	Brown anole	*Anolis sagrei*	1	0.6
Total		* *	**155**	

Blood meals determined by PCR-based assays using primers which targeted the 16s rRNA and cytochrome *b* genes for reptiles, amphibians, birds, and mammals. DNA sequences were identified to vertebrate host species using the National Center for Biotechnology Information (NCBI) GenBank Basic Local Alignment Search Tool (BLAST).

Calculations of the VIF initially showed correlation between environmental variables, especially landscape metrics, resulting in the evaluation of two separate candidate sets and the elimination of the developed medium habitat classification in the final set of variables. Two candidate variable sets were used in model runs, each set included the four bioclim variables and either the edge density habitat classes or the percent land cover habitat classes (Supplementary Table S2). All calculated VIF values were <5 for both candidate sets, indicating low correlations between layers used for model calibration.

A total of 48 models were generated using ENMeval across the two candidate sets of environmental variables. The best performing model in ENMeval was identified based on the lowest AICc value = 920.3, a high AUC of the receiver operating characteristic (ROC) (0.925) and a low difference in the AUC of the ROC of the internal training and testing data (0.057). The best model was generated using the “percent land cover” variable set (Table 2), a combination of “linear” + “product” feature classes, and a regularization multiplier value of 3. Results from the model run in the Maxent software program using the same parameter values had an AUC of the ROC value = 0.919. Four environmental variables and nine habitat classifications were retained in the best performing model (Table 2). Three of the four environmental variables contributed to 80% of the model performance, with maximum temperature of the warmest month contributing the most (31.6%), followed by minimum temperature of the coldest month (28.5%), and precipitation of the driest month (19.9%) (Table 2). Precipitation of the wettest month contributed the least to model performance of the four environmental variables (4.6%). In contrast, landscape variables including percent land cover contributed less to model performance, with six of the nine variables contributing <1.0% (Table 2). The three habitat classifications contributing the greatest to the model performance included emergent herbaceous wetlands (5.2%), woody wetlands (4.3%), and developed open (2.7%).

**Table 2:** Estimates of the relative contributions and permutation importance of each environmental and landscape variable used in the final model.

Variable	Percent contribution	Permutation importance
Max temperature of warmest month (bio5)	31.6	0.3
Min temperature of coldest month (bio6)	28.5	27.8
Precipitation of driest month (bio14)	19.9	3.9
Emergent herbaceous (percent land cover) (95)	5.2	6.2
Precipitation of wettest month (bio13)	4.6	4.1
Woody wetlands (percent land cover) (90)	4.3	4.8
Developed open (percent land cover) (21)	2.7	11.1
Mixed forest (percent land cover) (43)	0.9	0.3
Developed low (percent land cover) (22)	0.9	3.0
Cultivated crops (82)	0.8	0.9
Grassland/Herbaceous (percent land cover) (71)	0.5	4.4
Shrub/Scrub (percent land cover) (52)	0.1	1.0
Deciduous forest (percent land cover) (41)	0.1	0.3

**Figure 2:**
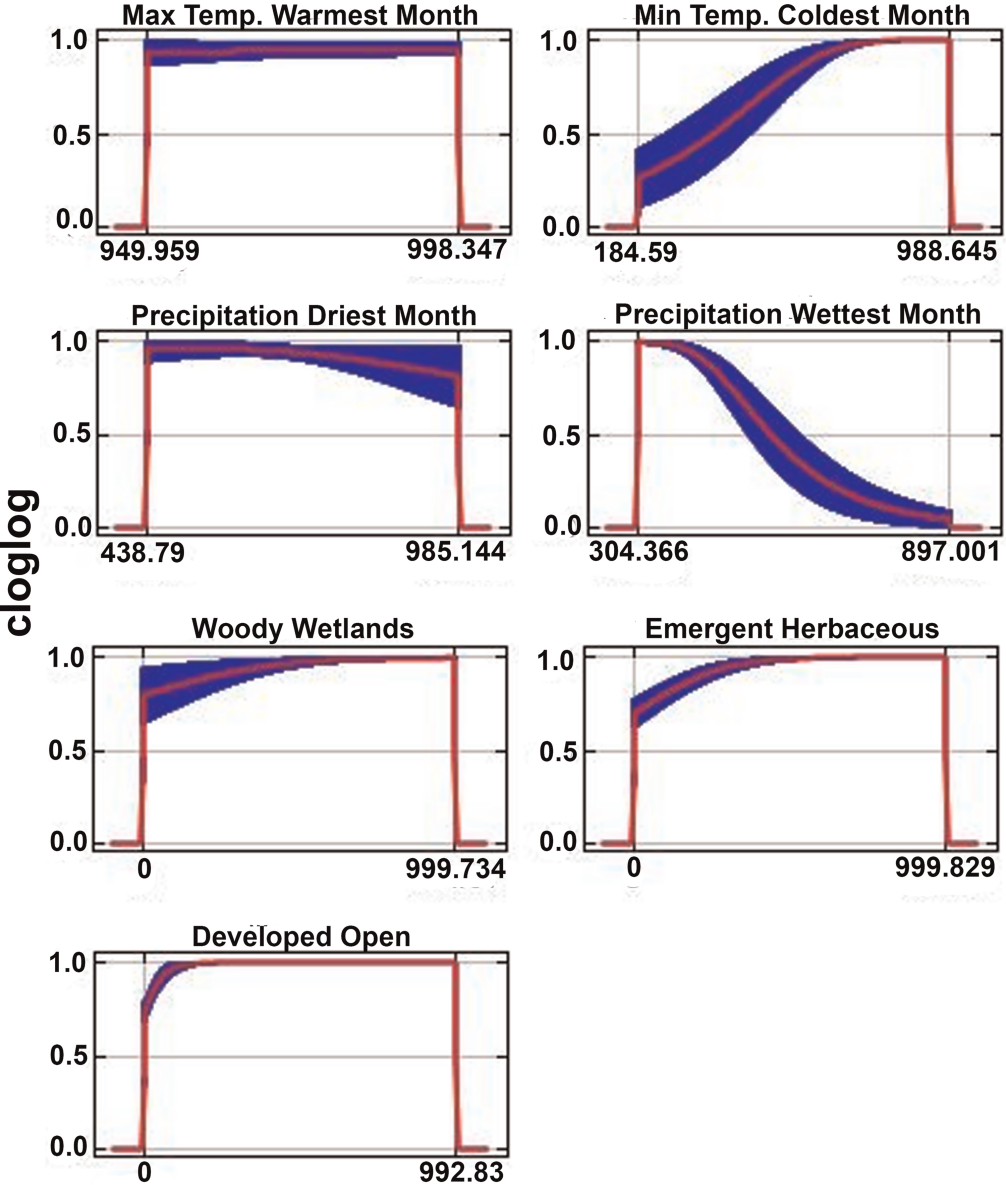
Response curves of bioclimatic and habitat classification variables used in the final model. X-axes represent standardized values of model variables. Only the seven variables contributing the most (>96%) to model performance were included in the figure.

For maximum temperature of the warmest month, the environmental variable with the greatest percent contribution, environmental suitability remained high across the entire range of maximum temperature of the warmest month (Figure 2). In contrast, environmental suitability generally increased as maximum temperature of the coldest month increased, with suitability tapering off at the highest temperatures (Figure 2). Response curves for precipitation of the driest month indicated a marginal decrease in suitability with increased precipitation during the driest month, whereas precipitation of the wettest month showed a slight decrease as precipitation decreased during the wettest month (Figure 2). The three habitat classifications contributing greatest to model performance included the emergent herbaceous wetlands, woody wetlands, and developed open landscapes (Table 2). Suitability values for these variables all showed slight increases as percent land cover increased for each of the classes (Figure 2).

**Figure 3:**
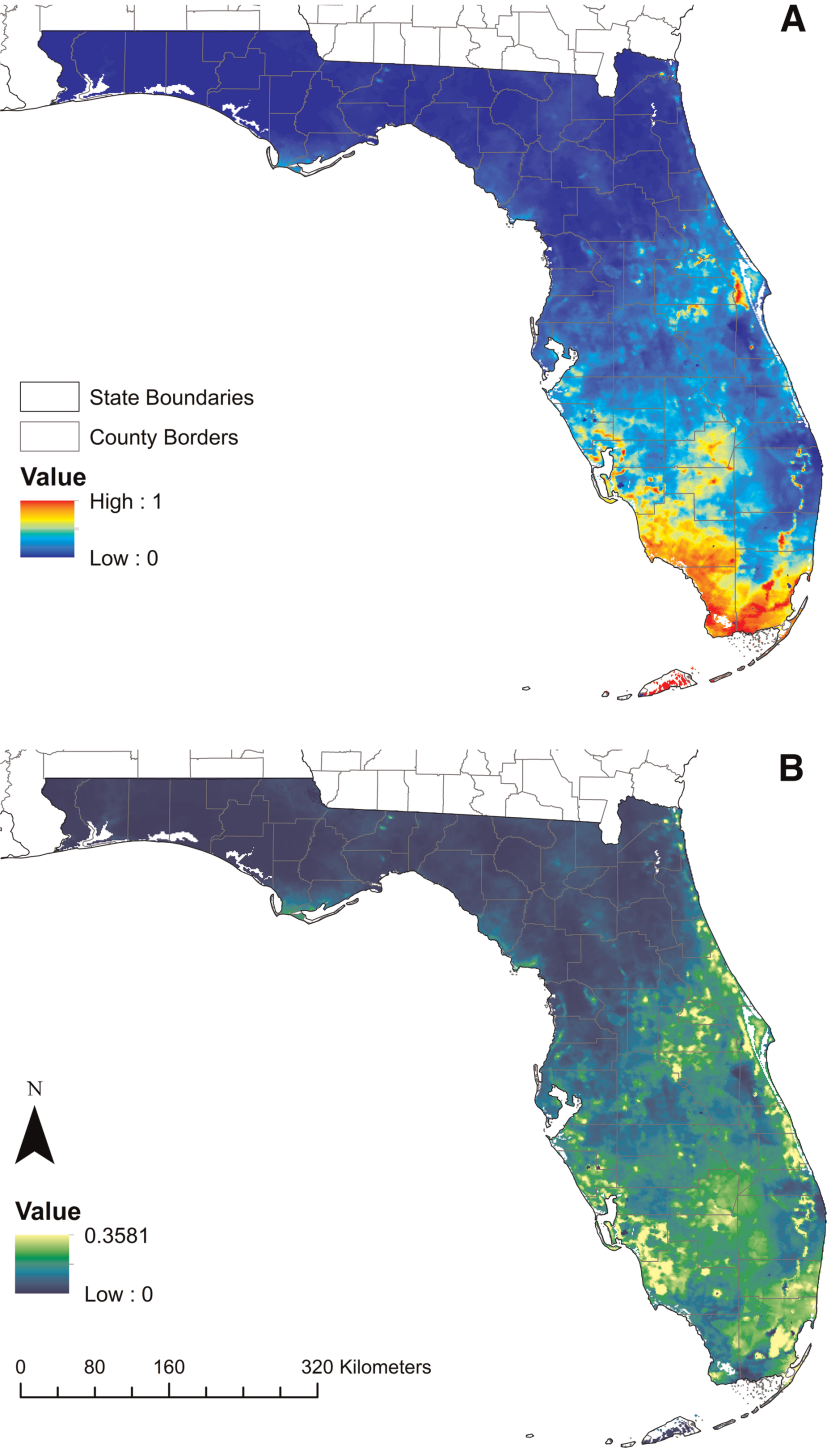
Model projections of the predicted distribution of *Cx. cedecei*/rodent host interactions in Florida. (**A**) Model predicting the distribution of Everglades virus vector/host interaction in Florida, USA including the calibration and projection region; red areas indicate high predicted suitability, while blue areas predict low suitability; (**B**) standard deviation of predicted suitability of the model across 50 bootstrap replicates; yellow areas show areas with higher standard deviation while blue areas indicate a lower standard deviation.

The model predicted that substantial areas of Florida are suitable for *Cx. cedecei* feeding upon rodents, with large suitable areas in the southern half of the Florida Peninsula, and smaller areas along the Atlantic Coast, the central peninsula, and the northern Gulf Coast (Figure 3). The area of highest predicted environmental suitability was observed in southernmost Florida, consisting of the Florida Keys, and the majority of Collier, Miami-Dade and Monroe Counties. Standard deviation values in these regions were low, indicating lower variability in model outputs and higher confidence in the predicted suitability in these areas (Figure 3). Areas of high suitability were also predicted throughout Lee County, Glades County, Highlands County, the western half of Charlotte County, and the southern half of Sarasota County, with areas of moderate suitability predicted throughout much of these counties (Figure 3). Although low suitability was predicted throughout the Miami Metropolitan Area (City of Miami to West Palm Beach), there were pockets of predicted high suitability on the western margins of these urban areas, where they reach the greater Everglades area (Figure 3). Standard deviation values were higher in these transitional areas, indicating lower model confidence for these areas (Figure 3). An area of patchy high suitability was observed west of Cape Canaveral, consisting of substantial portions of five counties (Brevard, Lake, Orange, Osceola and Seminole Counties) (Figure 3). Smaller low-to-moderate areas of suitability are also predicted for coastal portions of the entire Atlantic Coast, excluding the Miami Metropolitan area. Small areas of the Florida Panhandle were predicted to have low suitability for vector-host interactions (portions of Franklin, Gulf and Leon Counties) (Figure 3). These areas of low predicted suitability in the Panhandle had moderate standard deviation values, indicating relatively high variability in model confidence in these regions.

## Discussion

The objective of this study was to model areas where EVEV virus transmission could occur using georeferenced *Cx. cedecei* blood meals derived from rodent hosts, to investigate whether this method produces accurate models to predict the distribution of the nidus of transmission. The best performing model included four climate variables summarizing parameters for precipitation and temperature, and nine landscape variables using percent land cover data. Collectively, climate variables contributed to 84.6% of the model, while landscape variables contributed only 15.4% of the model. In general, areas with the greatest predicted suitability for *Cx. cedecei* feeding upon rodents included subtropical regions of southern Florida, as well as less-developed areas in central Florida (Figure 3). Other areas with high predicted suitability included areas inland from Cape Canaveral through Lake County, and along the Atlantic Coast (Figure 3).

Precipitation and temperature bioclimatic variables contributed the greatest to model performance, suggesting that climatic factors play an important role in constraining the distribution where *Cx. cedecei* encounters and feeds upon rodents. This is likely due to the way that water levels and temperature limits throughout the year influence both the subterranean larval habits of *Cx. cedecei* as well as the reproductive cycles of the vertebrate hosts of EVEV. For example, the model showed that suitability of *Cx. cedecei* feeding on rodent hosts increased with increased minimum temperatures during the coldest month, which corresponds to the subtropical portions of the state (Figure 2). Since the minimum temperature during the coldest month contributes a substantial amount to the performance of our model, it is understandable that much of southwestern Florida is considered suitable for *Cx. cedecei* to feed on rodent hosts, since these are where the warmest temperatures occur in Florida (59). This interpretation is supported by the finding that suitability for *Cx. cedecei* feeding upon rodents remained high, and even increased slightly, with increasing temperatures during the warmest month (Figure 2), which contributed the most to model performance (31.6%), indicating that *Cx. cedecei* feeds upon rodents in tropical areas of the state. Therefore, with potentially increased annual temperatures and changes in mean daily minimum and maximum temperatures predicted in the coming years due to climate change (60, 61), prevalence of suitable areas were *Cx. cedecei* feeds upon rodent hosts is likely to increase, especially in areas with similar woody wetland coastal habitats such as areas along the Gulf Coast including the Florida Panhandle, Louisiana, and the Texas coast. Finally, that suitability for *Cx. cedecei* to feed on rodent hosts decreased with increasing precipitation during the driest month (contributing to 19.9% of model performance) could be due to poorly defined wet and dry seasons of temperate (northern) Florida, where *Cx. cedecei* is not known to occur. However, that increased precipitation during the driest months would decrease suitability for vector/host interactions is somewhat surprising, considering that most EVEV amplification is thought to occur during the months of July through October (the height of the wet season) in the northern Everglades, when both hispid cotton rats and cotton mice are concentrated in hardwood hammocks of the Everglades (45). Because climate might be a primary driver of the phenology of both vector and host, it is predictable that precipitation and temperature variables had such high contributions to model performance (24, 45).

Landscape variables contributed considerably less than climate variables to the performance of the best model (Table 2). The landscape variables with the greatest contributions were percentage of emergent herbaceous wetland, which contributed 5.2% to model performance, and percentage of woody wetlands, contributing 4.6% (Table 2). Emergent herbaceous wetlands constitute marshes, which cover a large portion of the Greater Everglades Ecosystem, and during the dry season, are important reproductive habitats for the cotton rat and cotton mouse. Woody wetlands include mangrove forests, cypress swamps, and hardwood swamps, which are confirmed habitats of *Cx. cedecei*. *Culex cedecei* may encounter and feed upon muroid rodents where these habitat classes overlap, indicating that EVEV transmission may not be restricted to the hardwood hammocks of the southern Everglades ecosystem, as earlier studies indicated (15, 19, 23, 25, 45, 62). This is corroborated by Fish et al. (63) which reported EVEV isolations from mosquito pools from diverse habitats, including hardwood hammocks, mangroves, strand swamp, and pinelands. Everglades virus was detected in mosquitoes in all of these habitats in 2013 (a high-water year), but in only hardwood hammocks in 2014 (a low-water year), suggesting that hardwood hammocks may be primarily crucial for the maintenance of EVEV during dry periods, likely because hardwood hammocks provide subterranean refuge for larval *Cx. cedecei* in limestone solution holes and are the only available dry habitat accessible to rodents during periods of high water (35). Although the landscape variables used here did not contribute as much to model performance as expected, it is possible that additional habitat variables such as water table depth would have a more substantial impact on the model, considering the larval habitat of *Cx. cedecei* in many subterranean habitats such as solution-holes or crab burrows.

**Figure 4:**
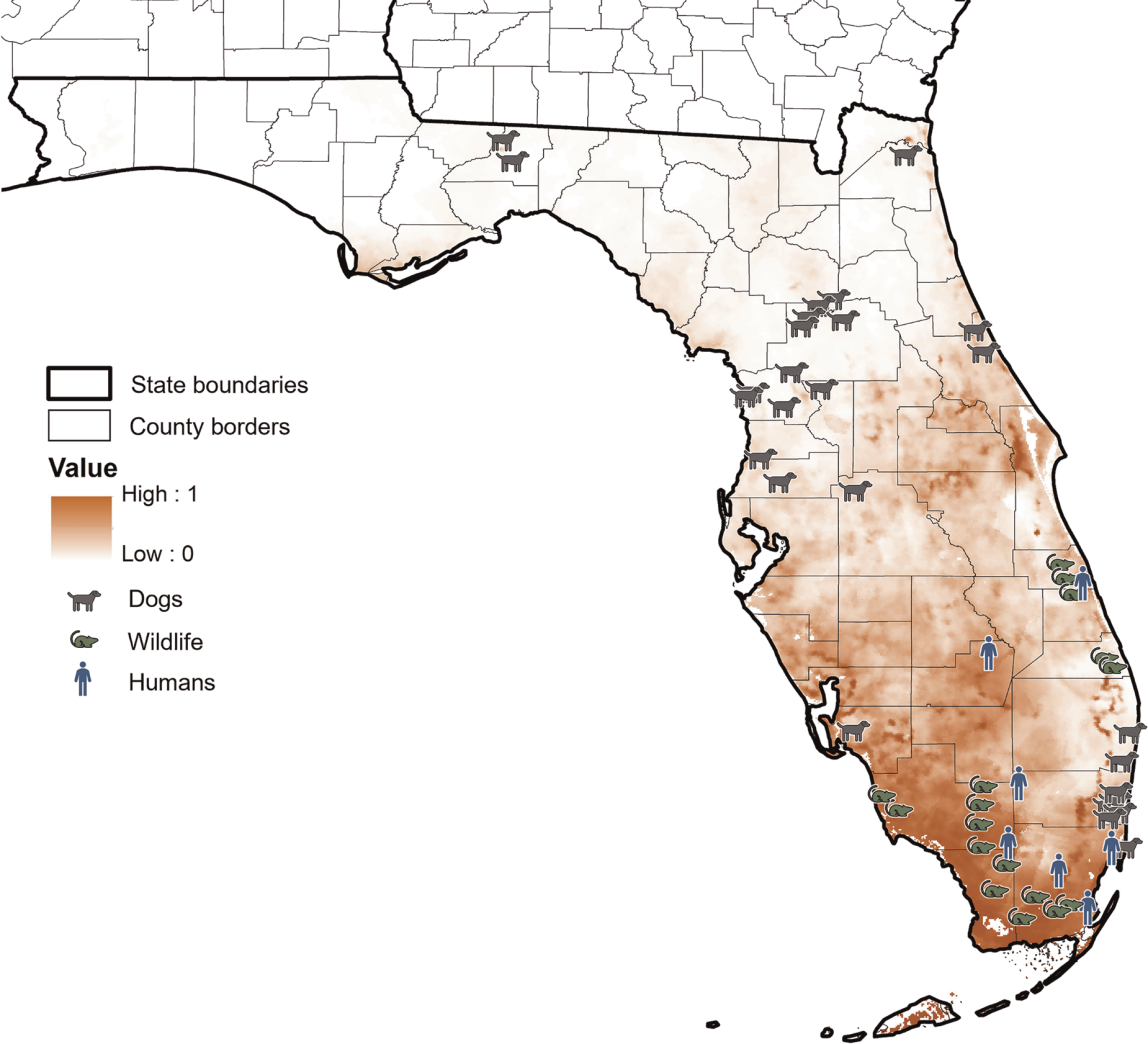
Model projections of the predicted distribution of *Cx. cedecei*/rodent host interactions in Florida, overlaid with historical detections of infections and antibodies to EVEV in Florida. Dark values represent higher probability of *Cx. cedecei*/rodent host interactions. Dog symbols represent locations where EVEV neutralizing antibodies were found in Florida dogs, rodent symbols represent locations where EVEV neutralizing antibodies or EVEV was detected in Florida wildlife, while human symbols represent human disease due to EVEV.

Areas predicted suitable for *Cx. cedecei* blood meals from rodents corresponded closely to locations of historical EVEV cases in humans and infections in wildlife, but not for a recent serosurvey of dogs (Figure 4). The seven locations where historical human cases and detection of antibody presence of EVEV recorded from 1960 to 1971 occurred in areas predicted to be moderate to highly suitable for *Cx. cedecei*/rodent interactions in Florida, supporting the notion that proximity to areas where *Cx. cedecei* actively feeds upon rodent amplifying hosts could lead to higher spillover risk to humans (30–33). In some areas, however, such as Vero Beach, Florida, where a 78-year-old man was diagnosed with EVEV in 1968, our models predict only moderately suitable environments for *Cx. cedecei*/rodent host interactions for EVEV (32). Vero Beach has undergone significant development since the 1960's, likely reducing suitable habitat for EVEV transmission since that period (64). Georeferenced data of antibody and virus detection in wildlife in Florida undertaken in the 1970's also show much overlap with areas predicted highly suitable for *Cx. cedecei* blood meals from rodent hosts (22, 24, 34), also supporting the importance of these organisms in transmission of EVEV. In contrast, the predicted distribution of *Cx. cedecei* blood meals from rodent hosts in Florida do not overlap substantially with serological evidence of EVEV exposure in sentinel dogs (36). Coffey et al. (36) speculate that other vector species may be involved in transmission of EVEV, however no other mosquito species in Florida feeds heavily upon rodents, the only recognized vertebrate hosts of EVEV (15, 65–69). Therefore, more work is needed to determine whether *Cx. cedecei* occurs outside of the regions predicted to be suitable for the presence of *Cx. cedecei* feeding on rodent hosts in this study to help to explain antibody detections in sentinel dogs in these areas. Although most previous studies suggest that the distribution of *Cx. cedecei* blood meals from rodent hosts are likely to be accurately represented by our model, future studies of arbovirus detection in other Florida mosquito and vertebrate populations may still be warranted to rule out the possibility of other vector and host species involvement in the transmission of EVEV.

In total, there were 14 locations where *Cx. cedecei* was collected but no rodent blood meals were detected, indicating that *Cx. cedecei* feeds readily upon nonrodents and host use of this vector is influenced by local environment and available mammals (16, 18). At most of these sites (13 of 14), insufficient sampling (two or fewer blood meals), may have affected the lack of rodent blood meals. However, given the naturally high fraction of blood meals that Cx. cedecei takes from rodents [>50% (15),], we expect that even with low sample sizes (*n* > 3) rodent blood meals would be detected. At one location in Polk County, three blood-engorged *Cx. cedecei* were analyzed, and two females fed upon cottontail rabbit while a third fed upon Virginia opossum, suggesting that even if *Cx. cedecei* does encounter rodents at this site, its feeding upon rodents could be too infrequent to support EVEV transmission.

This study has several limitations. Sampling was fairly opportunistic, relying heavily upon properties with access granted to mosquito control district personnel. More sampling, across more sites distributed across all landcover classes may have yielded additional insights into the environmental drivers of vector-host contact. The assumption that EVEV is maintained in nature solely by *Cx. cedecei* and rodent amplifying hosts, while supported by many studies (15, 65–70), is not supported by evidence from EVEV-seropositive dogs in areas of Florida that are far outside the known range of *Cx. cedecei* (36). There are also other aspects of virus transmission which are not considered in this model such as pathogen presence and detection, vector competency of varying populations of *Cx. cedecei*, and host competency of rodent populations and subspecies (71) (vector and host competence is known to change between populations of mosquito species and subspecies of hosts). Pathogen presence and detection was not undertaken as a part of this study primarily because we wished to model the potential areas where pathogen transmission might occur due to the interactions between vectors and vertebrate hosts. Furthermore, many thousands of mosquitoes (approximately 1,000 female mosquitoes per site) must be collected, identified and pool screened by virus isolation or PCR to sample the minimum number needed to detect EVEV in the vector, exponentially increasing the effort and expense. We show that with a small number of blood-engorged specimens per site, the conditions conducive for transmission can be modelled across the landscape. Including virus data may also limit risk estimates, and potentially introduce spatial sampling biases (12). Finally, in this effort, we did not compare predicted distributions of *Cx. cedecei* rodent bloodmeals with predicted distributions of vector or host species alone. Cotton rats have a ubiquitous distribution occupying a broad range of habitats in the study area and therefore model predictions may not be widely informative. However, Sloyer et al. (2022) presents an ecological niche model predicting the potential distribution of *Cx. cedecei* in Florida, which exhibits greater habitat constraints, and this model output provides a valuable resource for visual comparison.

In this study, we employ geographically referenced *Cx. cedecei* blood meals to investigate whether locations where vectors feed upon EVEV pathogen hosts can be used in ecological niche modeling to predict areas where the potential nidus of transmission may occur. This method should allow for better inference of potential transmission areas than models which only consider the vector or amplification host species individually. Comparing predicted distributions of *Cx. cedecei* feeding upon rodent hosts in the present study to historical human cases of EVEV disease, as well as antibodies in wildlife show substantial overlap with areas predicted moderate to high for these vector/host associations. As such, the findings of this study likely predict the most accurate distribution of the nidus of EVEV to date. A similar approach using host blood meals of other arboviruses can be used to predict potential areas of virus transmission for other vector-borne diseases such as eastern equine encephalitis virus, West Nile virus, or St. Louis encephalitis virus in the United States. Knowledge of areas where vectors feed on amplifying hosts of arboviruses can infer risk to potential human spillover of disease, drawing attention to areas where undiagnosed causes of encephalitis or acute disease in humans could be the result of arbovirus infection.

## Data Availability

The original contributions presented in the study are included in the article/**Supplementary Material**, further inquiries can be directed to the corresponding author.

## References

[1] Galuzo IG. Landscape epidemiology (epizootiology). Adv Vet Sci Comp Med. (1975) 19:73–96. 1108620

[2] Reisen WK. Landscape epidemiology of vector-borne diseases. Annu Rev Entomol. (2010) 55:461–83. 10.1146/annurev-ento-112408-085419 19737082

[3] Pavlovsky EN Pious FK. Natural nidality of transmissible diseases, with special reference to the landscape epidemiology of zooanthroponoses. Natural nidality of transmissible diseases, with special reference to the landscape epidemiology of zooanthroponoses (1966). Available at: https://www.cabdirect.org/cabdirect/abstract/19672203502 (Accessed June 2, 2022).

[4] Peterson AT. Mapping disease transmission risk: Enriching models using biogeography and ecology. Baltimore: JHU Press (2014).

[5] Baak-Baak CM Moo-Llanes DA Cigarroa–Toledo N Puerto FI Machain-Williams C Reyes-Solis G Ecological niche model for predicting distribution of disease-vector mosquitoes in Yucatán state, México. J Med Entomol. (2017) 54:854–61. 10.1093/jme/tjw243 28399263 PMC6503852

[6] Sloyer KE Burkett-Cadena ND Campbell LP. Predicting the potential distribution of Culex (Melanoconion) cedecei in Florida and the Caribbean using ecological niche models. J Vector Ecol. (2022) 47:88–98. 10.52707/1081-1710-47.1.88 36629360

[7] Gholamrezaei M Mohebali M Hanafi-Bojd AA Sedaghat MM Shirzadi MR. Ecological niche modeling of main reservoir hosts of zoonotic cutaneous leishmaniasis in Iran. Acta Trop. (2016) 160:44–52. 10.1016/j.actatropica.2016.04.014 27150212

[8] Blackburn JK Matakarimov S Kozhokeeva S Tagaeva Z Bell LK Kracalik IT Modeling the ecological niche of Bacillus anthracis to map anthrax risk in Kyrgyzstan. Am J Trop Med Hyg. (2017) 96:550–6. 10.4269/ajtmh.16-0758 28115677 PMC5361526

[9] León B Jiménez-Sánchez C Retamosa-Izaguirre M. An environmental niche model to estimate the potential presence of Venezuelan equine encephalitis virus in Costa Rica. Int J Environ Res Public Health. (2021) 18:227. 10.3390/ijerph18010227 PMC7795298 33396763

[10] Peterson AT Sánchez-Cordero V Ben Beard C Ramsey JM. Ecologic niche modeling and potential reservoirs for chagas disease, Mexico. Emerg Infect Dis. (2002) 8:662–7. 10.3201/eid0807.010454 12095431 PMC2730326

[11] Samy AM Campbell LP Peterson AT. Leishmaniasis transmission: distribution and coarse-resolution ecology of two vectors and two parasites in Egypt. Rev Soc Bras Med Trop. (2014) 47:57–62. 10.1590/0037-8682-0189-2013 24603738

[12] Johnson EE Escobar LE Zambrana-Torrelio C. An ecological framework for modeling the geography of disease transmission. Trends Ecol Evol. (2019) 34:655–68. 10.1016/j.tree.2019.03.004 31078330 PMC7114676

[13] Dobson A Cattadori I Holt RD Ostfeld RS Keesing F Krichbaum K Sacred cows and sympathetic squirrels: the importance of biological diversity to human health. Plos Med. (2006) 3:e231. 10.1371/journal.pmed.0030231 16729846 PMC1472550

[14] Weaver SC Ferro C Barrera R Boshell J Navarro J-C. Venezuelan equine encephalitis. Ann Rev Entomol. (2004) 49:141–74. 10.1146/annurev.ento.49.061802.123422 14651460

[15] Edman JD. Host-feeding patterns of Florida mosquitoes (Diptera: Culicidae) VI. Culex (Melanoconion)1. J Med Entomol. (1979) 15:521–5. 10.1093/jmedent/15.5-6.521 544824

[16] Hoyer IJ Blosser EM Acevedo C Thompson AC Reeves LE Burkett-Cadena ND. Mammal decline, linked to invasive Burmese python, shifts host use of vector mosquito towards reservoir hosts of a zoonotic disease. Biol Lett. (2017) 13:20170353. 10.1098/rsbl.2017.0353 28978755 PMC5665769

[17] Hoyer IJ Acevedo C Wiggins K Alto BW Burkett-Cadena ND. Patterns of abundance, host use, and Everglades virus infection in Culex (Melanoconion) cedecei mosquitoes, Florida, USA. Emerg Infect Dis. (2019) 25:1093–100. 10.3201/eid2506.180338 31107225 PMC6537747

[18] Burkett-Cadena ND Blosser EM Loggins AA Valente MC Long MT Campbell LP Invasive Burmese pythons alter host use and virus infection in the vector of a zoonotic virus. Commun Biol. (2021) 4:1–11. 10.1038/s42003-021-02347-z 34183751 PMC8239020

[19] Coffey LL Carrara A-S Paessler S Haynie ML Bradley RD Tesh RB Experimental Everglades virus infection of cotton rats (Sigmodon hispidus). Emerg Infect Dis. (2004) 10:2182–8. 10.3201/eid1012.040442 15663857 PMC3323382

[20] Aguilar PV Estrada-Franco JG Navarro-Lopez R Ferro C Haddow AD Weaver SC. Endemic Venezuelan equine encephalitis in the Americas: hidden under the dengue umbrella. Future Virol. (2011) 6:721–40. 10.2217/FVL.11.5 21765860 PMC3134406

[21] Sanmartín C Mackenzie RB Trapido H Barreto P Mullenax CH Gutiérrez E Lesmes C. Encefalitis equina venezolana en Colombia, 1967. [Venezuelan equine encephalitis in Colombia, 1967] (1973). Available at: https://iris.paho.org/handle/10665.2/10836 (Accessed May 2, 2022). 4265714

[22] Bigler WJ. Venezuelan Encephalitis antibody studies in certain Florida wildlife. Wildl Dis. (1969) 5:267–70. 10.7589/0090-3558-5.3.267 5817782

[23] Chamberlain RW Sudia WD Work TH Coleman PH Newhouse VF Johnston JG. Arbovirus studies in south Florida, with emphasis on Venezuelan equine encephalomyelitis virus. Am J Epidemiol. (1969) 89:197–210. 10.1093/oxfordjournals.aje.a120929 4387911

[24] Lord RD Calisher CH Sudia WD Work TH. Ecological investigation of vertebrate hosts of Venezuelan equine encephalomyelitis virus in south Florida. Am J Trop Med Hyg. (1973) 22:116–23. 10.4269/ajtmh.1973.22.116 4684882

[25] Bigler WJ Lewis AL Wellings FM. Experimental infection of the cotton mouse (Peromyscus gossypinus) with Venezuelan equine encephalomyelitis virus. Am J Trop Med Hyg. (1974) 23:1185–8. 10.4269/ajtmh.1974.23.1185 4429188

[26] Cameron GN Spencer SR. Sigmodon hispidus. Mamm Species. (1981) 158:1–9. 10.2307/3504057

[27] Barbour DB Humphrey SR. Status and habitat of the key largo woodrat and cotton mouse (Neotoma floridana smalli and Peromyscus gossypinus allapaticola). J Mammal. (1982) 63:144–8. 10.2307/1380680

[28] Washino RK Tempelis CH. Mosquito host bloodmeal identification: methodology and data analysis. Ann Rev Entomol. (1983) 28:179–201. 10.1146/annurev.en.28.010183.001143 6131641

[29] Fikrig K Harrington LC. Understanding and interpreting mosquito blood feeding studies: the case of Aedes albopictus. Trends Parasitol. (2021) 37:959–75. 10.1016/j.pt.2021.07.013 34497032

[30] Work TH. Serological evidence of Arbovirus infection in the seminole Indians of Southern Florida. Science. (1964) 145:270–2. 10.1126/science.145.3629.270 14171866

[31] Ehrenkranz NJ Sinclair MC Buff E Lyman DO. The natural occurrence of Venezuelan equine encephalitis in the United States. N Engl J Med. (1970) 282:298–302. 10.1056/NEJM197002052820603 5410814

[32] Ehrenkranz NJ Ventura AK. Venezuelan Equine encephalitis virus infection in man. Annu Rev Med. (1974) 25:9–14. 10.1146/annurev.me.25.020174.000301 4824504

[33] Ventura AK Buff EE JEN. Human Venezuelan equine encephalitis virus infection in Florida. Am J Trop Med Hyg. (1974) 23:507–12. 10.4269/ajtmh.1974.23.507 4823800

[34] Bigler WJ. Serologic evidence of Venezuelan equine encephalitis virus infections in raccoons of south central Florida. J Wildl Dis. (1971) 7:166–70. 10.7589/0090-3558-7.3.166 5156482

[35] Bigler WJ Jenkins JH. Population characteristics of Peromyscus gossypinus and Sigmodon hispidus in tropical hammocks of south Florida. J Mammal. (1975) 56:633–44. 10.2307/1379476

[36] Coffey LL Crawford C Dee J Miller R Freier J Weaver SC. Serologic evidence of widespread Everglades virus activity in dogs, Florida. Emerg Infect Dis. (2006) 12:1873–9. 10.3201/eid1212.060446 17326938 PMC3291350

[37] Burkett-Cadena ND Hoyer I Blosser E Reeves L. Human-powered pop-up resting shelter for sampling cavity-resting mosquitoes. Acta Trop. (2019) 190:288–92. 10.1016/j.actatropica.2018.12.002 30521803

[38] Reeves LE Holderman CJ Gillett-Kaufman JL Kawahara AY Kaufman PE. Maintenance of host DNA integrity in field-preserved mosquito (Diptera: Culicidae) blood meals for identification by DNA barcoding. Parasit Vectors. (2016) 9:503. 10.1186/s13071-016-1791-z 27629021 PMC5024527

[39] Sloyer KE Acevedo C Runkel AE Burkett-Cadena ND. Host associations of biting midges (Diptera: Ceratopogonidae: Culicoides) near sentinel chicken surveillance locations in Florida, USA. J Am Mosq Control Assoc. (2019) 35:200–6. 10.2987/19-6834.1 31647709

[40] McGregor BL Stenn T Sayler KA Blosser EM Blackburn JK Wisely SM Host use patterns of Culicoides spp. biting midges at a big game preserve in Florida, U.S.A., and implications for the transmission of orbiviruses. Med Vet Entomol. (2019) 33:110–20. 10.1111/mve.12331 30063255

[41] Blosser EM Stenn T Acevedo C Burkett-Cadena ND. Host use and seasonality of Culex (Melanoconion) iolambdis (Diptera: Culicidae) from eastern Florida, USA. Acta Trop. (2016) 164:352–9. 10.1016/j.actatropica.2016.10.001 27712940

[42] Hass CA Nussbaum RA Maxson LR. Immunological insights into the evolutionary history of caecilians (Amphibia: Gymnophiona): relationships of the Seychellean caecilians and a preliminary report on family-level relationships. Herpetol Monogr. (1993) 7:56–63. 10.2307/1466951

[43] Kitano T Umetsu K Tian W Osawa M. Two universal primer sets for species identification among vertebrates. Int J Legal Med. (2007) 121:423–7. 10.1007/s00414-006-0113-y 16845543

[44] Lee J Lee S Young JPW. Improved PCR primers for the detection and identification of arbuscular mycorrhizal fungi. FEMS Microbiol Ecol. (2008) 65:339–49. 10.1111/j.1574-6941.2008.00531.x 18631176

[45] Bigler WJ Ventura AK Lewis AL Wellings FM Ehrenkranz NJ. Venezuelan equine encephalomyelitis in Florida: endemic virus circulation in native rodent populations of Kverjfladcs hammocks. Am J Trop Med Hyg. (1974) 23:513–21. 10.4269/ajtmh.1974.23.513 4150911

[46] Carrara A-S Gonzales M Ferro C Tamayo M Aronson J Paessler S Venezuelan equine encephalitis virus infection of spiny rats. Emerg Infect Dis. (2005) 11:663–9. 10.3201/eid1105.041251 15890116 PMC3320368

[47] Barve N Barve V Jiménez-Valverde A Lira-Noriega A Maher SP Peterson AT The crucial role of the accessible area in ecological niche modeling and species distribution modeling. Ecol Modell. (2011) 222:1810–9. 10.1016/j.ecolmodel.2011.02.011

[48] Peterson AT Soberón J Pearson RG Anderson RP Martínez-Meyer E Nakamura M Ecological niches and geographic distributions (MPB-49). Princeton University Press. (2011) 49:328. 10.23943/princeton/9780691136868.001.0001

[49] Thornton MM Shrestha R Wei Y Thornton PE Kao S-C Wilson BE. Daymet: daily surface weather data on a 1-km grid for North America, version 4. ORNL DAAC (2020).

[50] Hijmans RJ Phillips S Leathwick J Elith J Hijmans MRJ. Package ‘dismo’. Circles. (2017) 9:1–68. https://cran.r-project.org/web/packages/dismo/index.html

[51] Dewitz J. National land cover database (NLCD) 2019 products (2021). 10.1016/j.rse.2016.12.026 PMC6657805 31346298

[52] Merow C Smith MJ Silander Jr JA. A practical guide to MaxEnt for modeling species’ distributions: what it does, and why inputs and settings matter. Ecography. (2013) 36:1058–69. 10.1111/j.1600-0587.2013.07872.x

[53] Naimi B. R package usdm: uncertainty analysis for species distribution models (2013). Available at: http://cran.r-project.org/web/packages/usdm/usdm.pdf.

[54] Pratt HD Wirth WW Denning DG. The occurrence of Culex opisthopus Komp in Puerto Rico and Florida, with a description of the larva (Diptera, Culicidae). Proc Ent Soc Wash. (1945) 47:245–51.

[55] Muscarella R Galante PJ Soley-Guardia M Boria RA Kass JM Uriarte M ENMeval: an R package for conducting spatially independent evaluations and estimating optimal model complexity for Maxent ecological niche models. Methods Ecol Evol. (2014) 5:1198–205. 10.1111/2041-210X.12261

[56] Kass JM Muscarella R Pinilla-Buitrago E Galante PJ. ENMeval 2.0.0. (2021). Available at: https://jamiemkass.github.io/ENMeval/articles/ENMeval-2.0.0-vignette.html.

[57] Warren DL Seifert SN. Ecological niche modeling in Maxent: the importance of model complexity and the performance of model selection criteria. Ecol Appl. (2011) 21:335–42. 10.1890/10-1171.1 21563566

[58] Phillips SJ Anderson RP Schapire RE. Maximum entropy modeling of species geographic distributions. Ecol Modell. (2006) 190:231–59. 10.1016/j.ecolmodel.2005.03.026

[59] Irizarry-Ortiz MM Obeysekera J Park J Trimble P Barnes J Park-Said W Historical trends in Florida temperature and precipitation. Hydrol Process. (2013) 27:2225–46. 10.1002/hyp.8259

[60] Lobell DB Bonfils C Duffy PB. Climate change uncertainty for daily minimum and maximum temperatures: a model inter-comparison. Geophys Res Lett. (2007) 34. 10.1029/2006GL028726

[61] Loarie SR Duffy PB Hamilton H Asner GP Field CB Ackerly DD. The velocity of climate change. Nature. (2009) 462:1052–5. 10.1038/nature08649 20033047

[62] Carrara A-S Coffey LL Aguilar PV Moncayo AC Da Rosa APAT Nunes MRT Venezuelan equine encephalitis virus infection of cotton rats. Emerg Infect Dis. (2007) 13:1158–65. 10.3201/eid1308.061157 17953085 PMC2828070

[63] Weaver SC Scherer WF Taylor CA Castello DA Cupp EW. Laboratory vector competence of Culex (Melanoconion) cedecei for sympatric and allopatric Venezuelan equine encephalomyelitis viruses. Am J Trop Med Hyg. (1986) 35:619–23. 10.4269/ajtmh.1986.35.619 3706626

[64] Fish D Tesh RB Guzman H da Rosa APAT Balta V Underwood J Emergence potential of mosquito-borne arboviruses from the Florida Everglades. Plos One. (2021) 16:e0259419. 10.1371/journal.pone.0259419 34807932 PMC8608345

[65] Dybas CL. Florida’s Indian River Lagoon: an estuary in transition. BioScience. (2002) 52:554–9. 10.1641/0006-3568(2002)052[0555:FSIRLA]2.0.CO;2

[66] Edman JD Taylor DJ. Culex nigripalpus: seasonal shift in the bird-mammal feeding ratio in a mosquito vector of human encephalitis. Science. (1968) 161:67–8. 10.1126/science.161.3836.67 5659128

[67] Edman JD. Host-feeding patterns of Florida mosquitoes I. Aedes, Anopheles, Coquillettidia, Mansonia and Psorophora. J Med Entomol. (1971) 8:687–95. 10.1093/jmedent/8.6.687 4403447

[68] Edman JD. Host-feeding patterns of Florida mosquitoes: III. Culex (Culex) and Culex (Neoculex). J Med Entomol. (1974) 11:95–104. 10.1093/jmedent/11.1.95 4828351

[69] Edman JD. Host-feeding patterns of Florida mosquitoes: IV. Deinocerites. J Med Entomol. (1974) 11:105–7. 10.1093/jmedent/11.1.105 4151311

[70] Edman JD Haeger JS. Host-feeding patterns of Florida mosquitoes V. Wyeomyia. J Med Entomol. (1977) 14:477–9. 10.1093/jmedent/14.4.477 24743

[71] Edman JD Webber LA Kale HW. Host-feeding patterns of Florida mosquitoes II. Culiseta. J Med Entomol. (1972) 9:429–34. 10.1093/jmedent/9.5.429 4404140

