# Supplementary material for: Predicting potential transmission risk of Everglades virus in Florida using mosquito blood meal identifications
Source: Front Epidemiol. 2022 Dec 2;2:1046679. doi: 10.3389/fepid.2022.1046679 (PMC10910907; doi:10.3389/fepid.2022.1046679)
Supplement: Supplementary file 1 [file Table1.docx]

Supplementary Table 1. Locations of georeferenced rodent host interactions of *Cx. cedecei*.

| County | Latitude | Longitude | Hispid cotton rat | Cotton mouse | Black rat | Source |
| --- | --- | --- | --- | --- | --- | --- |
| Orange | 28.38861 | -81.5244 | ● | ● |  | This study |
| Osceola | 28.08089 | -81.0707 |  | ● |  | This study |
| Martin | 27.15966 | -80.4181 | ● |  | ● | This study |
| Martin | 27.23492 | -80.279 | ● | ● |  | This study |
| Lee | 26.47051 | -82.1566 |  |  | ● | This study |
| Collier | 26.04799 | -81.7091 | ● | ● |  | This study |
| Collier | 26.01754 | -81.6329 |  |  | ● | This study |
| Collier | 25.99712 | -81.6996 |  |  | ● | This study |
| Brevard | 28.59507 | -80.8634 | ● | ● |  | This study |
| Highlands | 27.54194 | -81.5078 |  |  | ● | This study |
| Brevard | 28.3709 | -80.8506 | ● |  |  | This study |
| Brevard | 28.37934 | -80.7647 | ● | ● |  | This study |
| Collier | 26.08312 | -81.7405 | ● |  |  | This study |
| Orange | 28.47495 | -81.6110 | ● | ● |  | This study |
| Orange | 28.44394 | -81.5426 | ● | ● |  | This study |
| Indian River | 27.58705 | -80.3698 | ● |  |  | This study |
| Monroe | 25.14888 | -80.9233 | ● |  |  | Hoyer et al., 2019 |
| Monroe | 25.18247 | -80.8977 |  |  | ● | Hoyer et al., 2019 |
| Miami-Dade | 25.35667 | -80.8222 |  | ● |  | Hoyer et al., 2019 |
| Miami-Dade | 25.33194 | -80.8028 | ● |  |  | Hoyer et al., 2019 |
| Miami-Dade | 25.40003 | -80.6598 | ● |  |  | Hoyer et al., 2019 |
| Miami-Dade | 25.32292 | -80.8332 | ● |  |  | Hoyer et al., 2019 |
| Miami-Dade | 25.25392 | -80.7982 | ● | ● |  | Hoyer et al., 2019 |
| Miami-Dade | 25.44089 | -80.7838 | ● |  |  | Hoyer et al., 2019 |
| Miami-Dade | 25.30194 | -80.7989 | ● | ● |  | Hoyer et al., 2019 |
| Miami-Dade | 25.42356 | -80.6797 | ● |  |  | Hoyer et al., 2019 |
| Miami-Dade | 25.40231 | -80.6158 | ● |  | ● | Hoyer et al., 2019 |
| Miami-Dade | 25.33889 | -80.818 | ● |  |  | Hoyer et al., 2019 |
| Miami-Dade | 25.43222 | -80.7772 | ● |  |  | Hoyer et al., 2019 |
| Monroe | 25.19997 | -80.8743 | ● | ● | ● | Hoyer et al., 2019 |
| Miami-Dade | 25.4175 | -80.6389 | ● | ● |  | Hoyer et al., 2019 |
| Monroe | 25.7540 | -80.9269 | ● | ● | ● | Burkett-Cadena et al., 2021 |
| Monroe | 25.75515 | -80.9291 |  | ● |  | Burkett-Cadena et al., 2021 |
| Monroe | 25.7616 | -80.9192 | ● | ● | ● | Burkett-Cadena et al., 2021 |
| Monroe | 25.7468 | -80.9482 | ● | ● | ● | Burkett-Cadena et al., 2021 |
| Collier | 25.97835 | -81.3847 | ● |  |  | Burkett-Cadena et al., 2021 |
| Collier | 25.97939 | -81.4221 |  | ● | ● | Burkett-Cadena et al., 2021 |
| Collier | 25.9970 | -81.4117 | ● | ● | ● | Burkett-Cadena et al., 2021 |
| Collier | 26.00156 | -81.4119 | ● | ● | ● | Burkett-Cadena et al., 2021 |
| Collier | 26.20384 | -81.3504 | ● | ● | ● | Burkett-Cadena et al., 2021 |
| Collier | 26.20788 | -81.3635 | ● | ● | ● | Burkett-Cadena et al., 2021 |
| Collier | 26.16236 | -81.3464 | ● | ● |  | Burkett-Cadena et al., 2021 |
| Collier | 26.17116 | -81.3485 | ● | ● |  | Burkett-Cadena et al., 2021 |
| Miami-Dade | 25.41128 | -80.4992 | ● |  |  | This study |
| Miami-Dade | 25.47051 | -80.3710 | ● |  |  | This study |
| Miami-Dade | 25.40644 | -80.5222 | ● |  |  | This study |
| Miami-Dade | 25.39335 | -80.5006 | ● |  |  | This study |
| Miami-Dade | 27.58447 | -80.3721 | ● |  |  | This study |
