# Supplementary material for: Predicting potential transmission risk of Everglades virus in Florida using mosquito blood meal identifications
Source: Front Epidemiol. 2022 Dec 2;2:1046679. doi: 10.3389/fepid.2022.1046679 (PMC10910907; doi:10.3389/fepid.2022.1046679)
Supplement: Supplementary file 2 [file Table2.docx]

Supplementary Table 2. Variance Inflation Factor (VIF) values for bioclimatic variables and habitat classes using Edge Density and Percent landcover for the habitat classes. Habitat classes were obtained from the National Land Cover Database Class Legend and Description (NLCD), and corresponding variance inflation factors calculated.

| Candidate variable set | Class | Variable Code | Classification Description | VIF |
| --- | --- | --- | --- | --- |
| Edge Density | Developed | 21 | Developed Open | 4.284101 |
|  | Developed | 22 | Developed Low | 4.682982 |
|  | Forest | 41 | Deciduous Forest | 1.374412 |
|  | Forest | 43 | Mixed Forest | 1.288128 |
|  | Shrubland | 52 | Shrub/Scrub | 2.102843 |
|  | Herbaceous | 71 | Grassland/Herbaceous | 1.792809 |
|  | Planted/Cultivated | 82 | Cultivated Crops | 1.109062 |
|  | Wetlands | 90 | Woody Wetlands | 1.812750 |
|  | Emergent Herbaceous | 95 | Emergent Herbaceous Wetlands | 2.242187 |
|  | Temperature | bio5 | Max Temperature of Warmest Month | 1.541781 |
|  | Temperature | bio6 | Min Temperature of Coldest Month | 2.792155 |
|  | Precipitation | bio13 | Precipitation of Wettest Month | 1.258239 |
|  | Precipitation | bio14 | Precipitation of Driest Month | 2.065098 |
| Percent Land Cover | Developed | 21 | Developed Open | 2.517648 |
|  | Developed | 22 | Developed Low | 2.960850 |
|  | Forest | 41 | Deciduous Forest | 1.132145 |
|  | Forest | 43 | Mixed Forest | 1.180303 |
|  | Shrubland | 52 | Shrub/Scrub | 1.625710 |
|  | Herbaceous | 71 | Grassland/Herbaceous | 1.446197 |
|  | Planted/Cultivated | 82 | Cultivated Crops | 1.377836 |
|  | Wetlands | 90 | Woody Wetlands | 1.504654 |
|  | Emergent Herbaceous | 95 | Emergent Herbaceous | 1.884108 |
|  | Temperature | bio5 | Max Temperature of Warmest Month | 1.570331 |
|  | Temperature | bio6 | Max Temperature of Coldest Month | 2.685562 |
|  | Precipitation | bio13 | Precipitation of Wettest Month | 1.294220 |
|  | Precipitation | bio14 | Precipitation of Driest Month | 2.044670 |
